# A population-based controlled experiment assessing the epidemiological impact of digital contact tracing

**DOI:** 10.1038/s41467-020-20817-6

**Published:** 2021-01-26

**Authors:** Pablo Rodríguez, Santiago Graña, Eva Elisa Alvarez-León, Manuela Battaglini, Francisco Javier Darias, Miguel A. Hernán, Raquel López, Paloma Llaneza, Maria Cristina Martín, RadarCovidPilot Group, Oriana Ramirez-Rubio, Adriana Romaní, Berta Suárez-Rodríguez, Javier Sánchez-Monedero, Alex Arenas, Lucas Lacasa

**Affiliations:** 1 Member, Association for Computing Machinery (ACM), Barcelona, Spain; 2 Secretaría de Estado de Digitalización e Inteligencia Artificial (SEDIA), Secretaría General de Administración Digital, Ministerio de Asuntos Económicos y Transformación Digital, Madrid, Spain; 3 Dirección General de Salud Pública, Servicio Canario de la Salud, Gobierno de Canarias, Las Palmas, Spain; 4 Transparent Internet, DK-5370 Mesinge, Denmark; 5 Department of Epidemiology, Harvard TH Chan School of Public Health, Boston, MA USA; 6 Department of Biostatistics, Harvard TH Chan School of Public Health, Boston, MA USA; 7 Harvard-MIT Division of Health Sciences and Technology, Boston, MA USA; 8 User Experience, INDRA, Alcobendas, Spain; 9 Razona LegalTech, Madrid, Spain; 10 Centro de Coordinación de Alertas y Emergencias Sanitarias. Dirección General de Salud Pública, Calidad e Innovación. Ministerio de Sanidad, Madrid, Spain; 11 School of Journalism, Media and Culture, Cardiff University, Cardiff, CF101FS UK; 12 Departament d’Enginyeria Informática i Matemátiques, Universitat Rovira i Virgili, 43007 Tarragona, Spain; 13 School of Mathematical Sciences, Queen Mary University of London, London, E14NS UK; 14 Instituto de Física Interdisciplinar y Sistemas Complejos IFISC (UIB-CSIC), E-07122, Palma, Spain

**Keywords:** Viral infection, Computer science

## Abstract

While Digital contact tracing (DCT) has been argued to be a valuable complement to manual tracing in the containment of COVID-19, no empirical evidence of its effectiveness is available to date. Here, we report the results of a 4-week population-based controlled experiment that took place in La Gomera (Canary Islands, Spain) between June and July 2020, where we assessed the epidemiological impact of the Spanish DCT app Radar Covid. After a substantial communication campaign, we estimate that at least 33% of the population adopted the technology and further showed relatively high adherence and compliance as well as a quick turnaround time. The app detects about 6.3 close-contacts per primary simulated infection, a significant percentage being contacts with strangers, although the spontaneous follow-up rate of these notified cases is low. Overall, these results provide experimental evidence of the potential usefulness of DCT during an epidemic outbreak in a real population.

## Abstract (web summary)

While Digital contact tracing (DCT) has been argued to be a valuable complement to manual tracing in the containment of COVID-19, no empirical evidence of its effectiveness is available to date. Here, the authors report the results of a 4-week population-based controlled experiment, where they assessed the impact of the Spanish DCT app.

## Introduction

Digital contact tracing (DCT)^1^, i.e. using mobile phone apps to make contact tracing and notification between individuals, has recently been proposed to be a plausible complement of manual contact tracing^2–6^ within the Test, Trace and Isolate (TTI) containment strategy in the context of the COVID-19 pandemic^7–9^. While several countries, initially including Singapore or South Korea and more recently Switzerland, Italy, France or Germany to cite a few^10^ have started to deploy different implementations of such technology, to date there is however a lack of empirical evidence of the effectiveness of such DCT^11,12^. Indeed, putting the technical functionality of DCT apps aside, the effectiveness of digital solutions is largely unproven in real-world outbreak settings. Moreover, further concerns on the usefulness of DCT range from the possibility that the app detects avalanches of false close-contacts—what would subsequently overwhelm primary healthcare—to skepticism on the possible low adoption and adherence to the technology. Finally, privacy issues have also been flagged.

In this work, we aim to bridge this gap by designing and conducting a population-based controlled experiment to assess the technical and epidemiological viability of a DCT app. In May and June 2020 the Secretary of State of Digitalisation and Artificial Intelligence (SEDIA) developed a Spanish app called Radar COVID^13^, a bluetooth-mediated DCT technology based on the Apple/Google protocol^14^. The app embraces privacy-by-design and takes into account in its design the resulting impact on the citizens, technology and governance^15^. To further validate its usefulness in a controlled environment and anticipate its potential impact, during 4 weeks in July 2020 we conducted a controlled experiment in San Sebastián de La Gomera, a town of population 10,000 in La Gomera, Canary Islands (Spain). During this experiment, we were able to assess both the performance of the app and its epidemiological impact, according to a list of Key Performance Indicators (KPIs).

## Results

### Radar COVID app

Radar COVID is a bluetooth-based DCT app specifically designed for the COVID-19 pandemic based on the Exposure Notification System (ENS) developed by Apple and Google^14^. ENS, also called the Apple/Google protocol, is itself strongly influenced by DCT protocols such as the Decentralized Privacy-Preserving Proximity Tracing (DP3T^16^) or the Temporary Contact Number (TCN) protocol, but is directly implemented at the operating system level, thereby circumventing problems and allowing for more efficient operation as a background process, as well as ensuring interoperability between Android and iOS devices, which constitute the sheer majority of the market.

Radar COVID has been developed under the principles of privacy-by-design, embracing the goal of user anonymity and minimisation as prescribed by European legal standards and established in the GDPR^17^. Some of the main characteristics includes (i) lack of login or identification of any kind along the process; (ii) the user can remove or de-activate the app at any given time; (iii) interaction among mobile phones take place thanks to randomly generated ephemeral and privacy-preserving identifiers according to the DP3T protocol. (iv) The generated list of ephemeral identifiers is stored in a decentralised way for 14 days and subsequently destroyed.

The app then uses the Apple and Google API which is in charge of generating, managing and storing the daily list of ephemeral identifiers as well as the bluetooth-mediated interaction between mobile phones. Additionally, and in compliance with the DP3T protocol, a backend is deployed in a public cloud server to deal with the management of positive confirmed identifiers and additional functionalities, such as surveys. The app is coded in Kotlin (Android) and Swift (iOS) (for a detailed report of Radar COVID technical specifications and open-source code, see ref. ^13^).

**Fig. 1:**
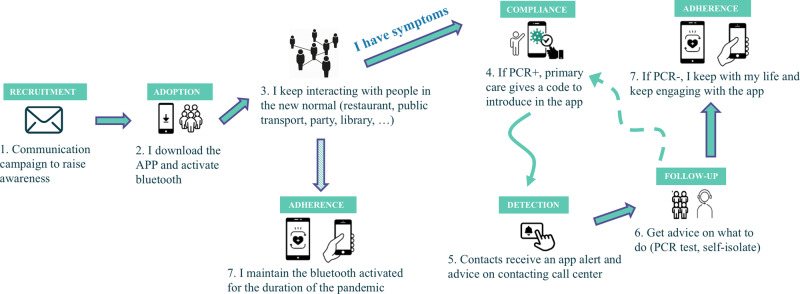
Radar Covid’s user journey. Describes the whole user journey, from the moment the user downloads the app until a close-contact is being notified by the app and what to do next.

The user journey is schematically depicted in Fig. 1. Once users downloaded the app, every phone starts to periodically transmit (via Bluetooth) ephemeral identifiers to its close physical neighbourhood. If another phone is close enough, it will receive the signal and establish an interaction. When two phones interchange ephemeral identifiers (i) for a duration larger than a certain threshold (15 min) and such that the Bluetooth attenuation suggests that the interaction happens below a certain physical distance (2 m), then a ‘match’ between the two phones is established, and this is the proxy for detecting a close-contact between the users. This match is stored by the API in each phone. In the event an individual is found to be infected (positive result of a PCR test), she is also provided at the point of care with a 12-digit code which she voluntarily introduces in the app. This code triggers the process of the app alerting her list of matches that they have been in close-contact with an infected individual.

### The controlled experiment

We designed and conducted a 4-week longitudinal population-based experiment with the aims of testing and validating, in a controlled environment, two different aspects of the Radar COVID app: its technical viability (i.e. whether the technology works in a realistic environment) and its epidemiological impact (i.e. whether the app can be useful to perform contact tracing and help contain an epidemic outbreak). The experiment was approved by the Public Health General Directorate of the Ministry of Health, Government of Spain.

The strategy to assess both tasks is based on initially propagating a thread of simulated infections across the population, then allowing people to freely interact while the “ infected” individuals are in the “asymptomatic” or “pre-symptomatic” phase, and subsequently monitoring how many of these simulated infections give rise to detection of close-contacts through the contact tracing app and the turnaround times of response to notifications.

The experiment took place between 29th June and 22nd July 2020 in San Sebastián de la Gomera, the capital and a municipality of La Gomera in the Canary Islands, Spain. The location was decided based on three criteria: (i) it is an island (i.e. easy management of intake), (ii) which is in a low-incidence state, and (iii) where different mobility patterns coincide (local population, tourists and commuters from other islands). Its population is ~10,000, including residents and daily commuters and the municipality also hosts the main harbour of the island. Downloading of RadarCovid app was voluntary and all participants provided an informed consent. The first days were devoted to marketing and communication aspects, such as highlighting the app in TV and local media, distribution of promotional material, etc. By the 10th of July—date of the first simulated outbreak—we expected a large percentage of the adoption cohort had already downloaded the app, either directly from the Apple/Google servers or with the help of marketing promoters and other venues. In the following weeks, several outbreaks were simulated, such that roughly 10% of the cohort that adopted the technology was infected, and digitally mediated detection of close-contacts was subsequently monitored. Such attack rate is in the order of magnitude of those that have been documented in a Singapore call centre (8.5%^18^) or in the Diamond Princess (18.8%^19^). We simulated a total of four epidemic outbreaks (three downtown, one in the Navieras—boats connecting La Gomera to Tenerife island) to explore different parametrisations: different asymptomatic times, Bluetooth calibrations (see Supplementary notes 2 and 3 for details) and different mobility patterns (we made sure that, across different outbreaks, each infected individual was only flagging an alert at most once). In retrospect, the adequate Bluetooth attenuation range for a correct implementation of the 15 min—2 metre rule was incorporated after the 15th July recalibration.

Both the analysis and dynamical tracking of the experiment was carried out based on data retrieved from the server, from extensive (voluntary and anonymous) app surveys, and from (voluntary and anonymous) follow-up calls of individuals to local primary health call centre, always on compliance with the privacy-by-design approach (see Supplementary note 4 for additional details).

### Key performance indicators

To assess the potential usefulness of the app in a realistic epidemiological context, we have defined seven KPIs against which we evaluated the experiment: five assessing the user behaviour (adoption, adherence, compliance, turnaround time, follow-up), and two assessing the effectiveness (overall detection, hidden detection). We consider that all these seven KPIs must be met satisfactorily in order for the app to be deployed:

- Adoption: This quantifies the percentage of the population that downloads the app, and provides an upper bound to the degree of penetration of the technology. Recently, the debate on contact tracing apps has gone through the misunderstanding of the results of an article^20^ which induced the message that a 60% target of adoption was needed for DCT to be effective. This seems a difficult target to achieve as a large percentage of the population might be skeptic and unwilling (or unable) to download the app. However, a deeper reading of the study reveals that the apps are already efficient at vastly lower levels of adoption. Given the non-linear relationship between adoption and mitigation of the disease, and the high heterogeneity of social networks, recent studies^4,6^ indeed show that levels of adoption above 20% already have an epidemiological impact in the containment strategy and thus justify their nationwide deployment.
- Adherence: Defined as the percentage of the population that has downloaded the app and still uses it 10 days after downloading it. In order for the technology to be disruptive, individuals need to both adopt it and adhere to it. We estimate this KPI indirectly via satisfaction surveys and number of active apps (from the public server).
- Compliance: Defined as the percentage of infected individuals that declare their status to the app by properly introducing a 12-digit code.
- Turnaround time: It considers two factors: the percentage of those infected individuals that declare their status to the app within the first 24 hours, and the average time between a code is introduced by a primary infection and a close-contact who receives an alert in the app makes the follow-up call to primary care to confirm the potential infection. The shorter the turnaround, the better we can minimise tracing delays, a critical parameter in the surveillance and containment of the epidemic^21,22^.
- Follow-up: Defined as the percentage of those individuals which, having been alerted by the app that they have been in close-contact to a PCR-positive (PCR+) case, decide to follow-up with a call to primary healthcare call centre. Whereas app users that receive an alarm can already voluntarily self-isolate or at least change their behaviour, this follow-up procedure is necessary to enable the contact to discuss her situation with a health professional who will eventually conduct the PCR test and provide advice.
- Overall detection rate: Defined as the average number of close-contacts of a given infected individual which are notified by the app. Ideally, this number is around or larger than the average number of close-contacts effectively traced by a manual contact tracer, although in general both digitally traced contacts do not necessarily need to fully overlap with manually traced contacts, and as such both tracing is complementary. In Spain, the median of manually traced contacts is about 3 (see Table 6 from^23^ and SI).
- Hidden detection: Defined as the percentage of the digitally traced close-contacts which are indeed unknown to the infected individual, and hence could constitute potential seeds for hidden transmission chains which by definition cannot be detected by manual contact tracing. This is a particularly important metric in regions where there is strong mixing, e.g. in touristic countries. We estimated this percentage via app surveys follow-up calls. While to the best of our knowledge there is no direct estimate in the literature of the percentage of close-contacts that an individual has with strangers to compare to, as a proxy we can use Polymod social contact network data where contacts are categorised as taking place at home, work, school or ‘other’^24,25^: we assume that most of the close-contacts that happen at ‘other’ locations typically happen among strangers (public transport, gym, library, restaurant, etc). Under this assumption, the percentage of hidden close-contacts would fluctuate across countries somewhat in the range 25–40%.

#### Implications of privacy-by-design

Since Radar COVID embraced a privacy-by-design approach, the data that could be retrieved from the API to analyse the KPIs was limited, and indirect evidence had to be sought via extensive follow-ups and online surveys, which nonetheless were always anonymous and privacy-preserving (see Supplementary note 4 for details). For the same reason, the numerical estimates of some KPIs are only approximate and we cannot provide the standard dispersion metrics which would be otherwise available with a more intrusive app. For instance, the estimated number of digitally traced contacts per primary infection is an indirect average estimate, we could not sample the whole distribution and accordingly do not have dispersion statistics. To further assess the efficiency of the app, in this experiment we were also able to measure an aggregated and anonymous metric quantifying the total daily number of notified alerts. Note that such metric is made available during the controlled experiment for the purpose of validating the efficiency of the app, and collected for research purposes as part of the privacy policy.

### Estimation of KPIs

In Table 1 we summarise the results of the experiment against all seven KPIs.

**Table 1:** Summary of key performance indicators and results of the population-based controlled experiment.

KPI	Result
Adoption	~33%, potentially larger based on indirect survey data.
Adherence	high during the whole duration of the experiment.
Compliance	64% of those cases that are given a code introduce it in the app.
Turnaround time	98% of those index cases that comply introduce the code within 24 h, and on average it takes 2.35 days between a simulated index case introduces a code in the app and the alerted close-contacts follow-up with call centre.
Follow-up	10% of notified close-contacts follow-up with a call to the designated point of care (call centre).
Overall detection	on average and after adequate Bluetooth calibration, the app can trace 6.3 close-contacts per index case.
Hidden detection	between 23% and 39% (depending on the survey form) of exposed close-contacts are strangers to the index case.

First, on relation to adoption, note that we could not use the number of downloads directly from the Apple and Google online stores (over 61k during the course of the experiment) as these are not geolocalised. Using indirect methods we estimate a 33% adoption, only using the amount of verifiable downloads directly performed offline by promoters, downloads from the Canary island government, and assuming a 2% spontaneous adoption percentage and a few other assumptions. This percentage could actually be just a lower bound as the spontaneous adoption could actually be larger, and in-depth interviews indeed suggest a much larger adoption estimate (see Supplementary note 5 for details). We shall also highlight that not having a more accurate estimate on adoption is a consequence of the app having followed a privacy-by-design approach.

While it is difficult to evaluate the adherence—as this metric suffers from the same privacy-by-design measurement problems than adoption—indirect evidence suggests this is likely to be high. First, according to the public server we recorded that over 12,000 apps were active throughout the duration of the experiment (with the capacity of receiving alert notifications), and this number was slightly increasing (see Supplementary note 6). We also gathered indirect evidence of a potentially high adherence from survey data: from 735 app surveys, 82% concluded that the app was a useful tool, and the question “I will recommend friends and family members to download and use Radar COVID” was given 7.8/10 marks. From the list of in-depth interviews (15 surveys) the question “I will keep on using the app when it is officially launched” reaches full marks. Note, however, that survey results on both adoption and adherence should be taken with caution and these might suffer from a selection bias (participants engaging with the experiment might be overly enthusiastic about the whole population’s engagement).

In relation to compliance, we inserted a total of 349 simulated infections: 119 among public workers, 181 among those who interacted with the app promoters, and the rest among visitors from Tenerife who came to the island by boat. An additional 43 secondary-case infections were given a code. Out of these 349 initial simulated infections, a total of 213 12-digit codes were introduced in the app, whereas 38 12-digit codes (of 43) were introduced as secondary cases. Combining both, we find an overall compliance of about 64% (61% for primary infections and 88% of secondary infections).

Some studies have recently emphasised the critical importance of a quick turnaround time for the epidemiological success of DCT^21,22^. In our study, about 98% (see Supplementary note 8 for details) of those who comply and introduce a code do so on time (within the first 24 hours), suggesting an initial very quick turnaround time. We then consider the average time from introducing the code in the app to the follow-up call that the close-contact makes to the designated point of care, to confirm whether the alert is really indicative of a potential infection. The average time across the different four outbreaks was 2.35 days, to be compared to the average of 2.6 days from onset of symptoms to isolation which is reported in manual contact tracing^26,27^.

Overall detection of secondary cases, estimated as the quotient of all cumulated new alerts over all cumulated introduced codes within a certain time window, is also significant. While initially we found an average of about 1.5 close-contacts per simulated primary case, after a proper recalibration of the matching parameters of the app (the Bluetooth parameters) that took place on the 15th of July, such overall detection rate increased up to an average of 6.3 close-contacts per primary case. Reaching such levels of detection is an indirect evidence that adoption density is high. Note that this is an average quantity and, again, the privacy-friendly design of the app makes it difficult to compute any dispersion estimate (e.g. standard deviation or interquartile range), because we cannot trace back the number of alerts to any particular infected individual: we only count how many daily alerts are triggered and how many codes are introduced. Accordingly, we cannot quantify the role or presence of superspreaders and the heterogeneity of the contact network. This uncertainty notwithstanding, our estimate after Bluetooth recalibration is almost twice as large as the median of manually detected contacts by professional contact tracers in Spain, which was about 3^23^ during the time of the experiment (note also that in July, Spain was overall in a very low-incidence state and thus manual contact tracing was efficient, so we expect the improvement of DCT over manual tracing to be even higher during an epidemic outbreak, where if not enough manual tracers are hired, the average number of manually traced contacts per primary case can be severely reduced, see Supplementary note 9). At the same time, the number of digitally detected contacts is manageable in the follow-up process, indicating that primary care is not overwhelmed by potential avalanches of false positives.

Hidden detection is also significant: 23% of the secondary cases that followed-up with primary care stated that the possible contagion probably happened with a stranger, and this number increases up to 39% according to the app survey (see Supplementary note 10 for details). Note that since this KPI is solely estimated based on questionnaires and surveys, we should treat it with extra caution, and we acknowledge that a more direct estimation is needed.

Finally, only about 10% of all potential secondary cases followed-up with primary care, i.e. yielding an effective average of 6.3 ⋅ 0.1 = 0.63 follow-up calls to the primary healthcare call centre per code introduced, or 0.64 ⋅ 6.3 ⋅ 0.1 = 0.4 follow-up calls per simulated infection.

## Discussion

Overall results of the controlled experiment study are positive and we can conclude that, a priori, this technology works and after appropriate communication campaigns it might have the sufficient level of penetration and compliance to help and serve as a useful complement to manual contact tracing and other non-pharmaceutical interventions in the containment of epidemic outbreaks, thus justifying its nationwide deployment. In particular, the fact that adoption is above threshold and that DCT not only seems able to detect more secondary cases than manual tracing but that a significant percentage of these could even contact with strangers is encouraging. Some additional comments and reflections on some challenges and limitations of such deployment are now in order.

Due to privacy-preserving issues, accurate estimation of the KPIs was difficult, and gauging their uncertainty was not always possible. Accordingly, our results, while very promising, should be treated with caution. As we have seen in the experiment, a successful adoption of the technology is underpinned by a substantial communication campaign. Also, we should distinguish overall adoption (the total percentage of the population that downloaded the app) from adoption density (which could vary from region to region within a country). For the DCT to work in a certain region, reaching sufficient adoption density is a necessary condition. This fact emphasises the importance of deploying not only strong nationwide communication campaigns, but also regional campaigns which aim at increasing local adoption densities.

One of the common concerns raised by the healthcare sector on relation to DCT is whether the app could trigger avalanches of false close-contacts—leading to an avalanche of false positives that could overwhelm primary healthcare resources. In this controlled experiment we haven’t observed such avalanches. While it is true that infections in this experiment were simulated—and thus should be further evaluated in real scenarios of disease transmission—the average number of matches per simulated primary case (6.3) was on the same order of magnitude than the average total number of contacts found in the BBC and Polymod data^24,25,28^. This suggests that in the context of this experiment the app is detecting about the correct amount of close-contacts and thus suggests large avalanches of false positives overwhelming primary healthcare would be unlikely.

Note that whereas Radar COVID is a nationwide DCT app, the Spanish healthcare system is decentralised, and competences are transferred to each autonomous community. Accordingly, an adequate deployment and operationalisation of the technology requires that each autonomous community integrates its healthcare system with the app. For instance, each autonomous community needs to be able to provide the 12-digit codes to PCR-positive cases in an agile and efficient way. Similarly, the follow-up system which takes place once close-contacts are alerted is unique for each autonomic healthcare system as well. Such integration is a critical factor underpinning success and has not been validated in this experiment.

While adoption and detection are high, the low percentage of those close-contacts that follow-up (10%) is concerning, and this is probably an important point to consider in any communication campaign devoted to raise awareness on the DCT app. We cannot distinguish whether this low percentage is due to the fact that in our experiment infections were simulated (so that users were aware that follow-up calls to the designated point of care would not lead in this case to PCR test and/or further assessment), or otherwise is due to the aversion of the user to proactively collaborate and make the call. Accordingly, further sociological studies should be carried out to investigate whether the fact that the close-contact is alerted by the app to have had a high-risk exposure at least helps to induce a modification on her behaviour (e.g. inducing spontaneous self-isolation), effectively reducing her contacts with others over some time window. What is however clear from our study is that if our estimation of the follow-up percentage is representative, then the ability of the app to detect tertiary or quaternary cases is seriously compromised, as no possibility of ‘cascading’ emerges.

Another source of uncertainty is long-term adherence. Whereas adherence was shown to be high in this study, we shall recall that the experiment only covered four weeks, an arguably short duration with respect to the real duration of the pandemic (at least several months until a vaccine can be deployed). Long-term adherence therefore remains unknown.

We should again highlight that in this controlled experiment infections are simulated, and this is a general limitation: since people in La Gomera are aware of this fact, we cannot extract any ‘behavioural’ conclusion of this study—e.g. we could not conclude whether those people that have downloaded the app are more or less risk averse. What is in any case clear is that the technology of Radar COVID works correctly and that if the results of the experiment are representative, then the nationwide deployment of the DCT app and its integration with the healthcare system of each autonomous community in Spain is justified and can contribute to the management and containment of the epidemic. In more general terms, this represents a much needed empirical evidence on the usefulness of DCT as a complementary nationwide epidemiological tool for the containment of COVID-19.

### Reporting summary

Further information on research design is available in the Nature Research Reporting Summary linked to this article.

## Supplementary information

Supplementary Information

Reporting Summary

## Data Availability

All data analysed during this study are included in this published article (and its supplementary information files).

## Code availability

No specific codes were developed to conduct the data analysis. Code and documentation of the app RadarCovid is available at^13^.

## References

[1] Farrahi K, Emonet R, Cebrian M (2014). Epidemic contact tracing via communication traces. PloS ONE.

[2] Ferretti L (2020). Quantifying SARS-CoV-2 transmission suggests epidemic control with digital contact tracing. Science.

[3] Ivers, L. C. & Weitzner, D. J. Can digital contact tracing make up for lost time? *Lancet Public Health* **5**, e417–e418 (2020) 10.1016/S2468-2667(20)30160-2 PMC7365621 32682488

[4] Bianconi, G., Sun, H., Rapisardi, G. & Arenas, A. A message-passing approach to epidemic tracing and mitigation with apps. *Phys. Rev. Res.* (in press).

[5] Aleta A (2020). Modelling the impact of testing, contact tracing and household quarantine on second waves of COVID-19. Nat. Hum. Behav..

[6] MorenoLópez, J. A. et al. Anatomy of digital contact tracing: role of age, transmission setting, adoption and case detection. *medRxiv*10.1101/2020.07.22.20158352 (2020). 10.1126/sciadv.abd8750 PMC8034853 33712416

[7] Zhu N (2020). A novel coronavirus from patients with pneumonia in China, 2019. N. Engl. J. Med..

[8] Huang C (2020). Clinical features of patients infected with 2019 novel coronavirus in Wuhan, China. Lancet.

[9] Hellewell J (2020). Feasibility of controlling COVID-19 outbreaks by isolation of cases and contacts. Lancet Global Health.

[10] https://www.technologyreview.com/2020/05/07/1000961/launching-mittr-covid-tracing-tracker/ (2020). Accessed 25 August 2020.

[11] Braithwaite I, Callender T, Bullock M, Aldridge RW (2020). Automated and partly automated contact tracing: a systematic review to inform the control of COVID-19. Lancet Digital Health.

[12] Anglemyer, A. et al. Digital contact tracing technologies in epidemics: a rapid review. *Cochrane Database Syst. Rev.*10.1002/14651858.CD013699 (2020). 10.1002/14651858.CD013699 PMC8241885 33502000

[13] The open-source code and documentation of Radar COVID is available at https://github.com/radarcovid

[14] Exposure Notifications: Helping fight COVID-19—Google. https://www.google.com/intl/en_us/covid19/exposurenotifications/

[15] Vinuesa, R., Theodorou, A., Battaglini, M & Dignum, V. A socio-technical framework for digital contact tracing. *Results Eng.* **8**, 100163 (2020). 10.1016/j.rineng.2020.100163 PMC7453267 38620324

[16] Troncoso, C. et al. Decentralized privacy-preserving proximity tracing. Preprint at https://arxiv.org/abs/2005.12273 (2020).

[17] Mobile applications to support contact tracing in the EU’s fight against COVID-19, eHealth Network, European Commission. https://ec.europa.eu/health/sites/health/files/ehealth/docs/covid-19_apps_en.pdf (2020).

[18] Park SY (2020). Coronavirus Disease Outbreak in Call Center, South Korea. Emerg. Infect. Dis..

[19] Yamahata Y, Shibata A (2020). Preparation for Quarantine on the Cruise Ship Diamond Princess in Japan due to COVID-19. JMIR Public Health Surveill..

[20] Hinch, R. et al. Effective configurations of a digital contact tracing app: a report to NHSX. https://github.com/BDI-pathogens/covid-19_instant_tracing/blob/master/Report (2020).

[21] Kretzschmar ME (2020). Impact of delays on effectiveness of contact tracing strategies for COVID-19: a modelling study. Lancet Public Health.

[22] Larremore, D. B. et al. Test sensitivity is secondary to frequency and turnaround time for COVID-19 screening. *Sci. Adv.* **7**, eabd5393 (2020). 10.1126/sciadv.abd5393 PMC7775777 33219112

[23] Informe COVID-19, ISCIII https://www.isciii.es/QueHacemos/Servicios/VigilanciaSaludPublicaRENAVE/EnfermedadesTransmisibles/Documents/GRIPE/Informe%20n%C2%BA%2038.Situaci%C3%B3n%20de%20COVID-19%20en%20Espa%C3%B1a%20a%206%20de%20agosto%20de%202020.pdf (2020).

[24] Prem K, Cook AR, Jit M (2017). Projecting social contact matrices in 152 countries using contact surveys and demographic data. PLoS Comput. Biol..

[25] Mossong J (2008). Social contacts and mixing patterns relevant to the spread of infectious diseases. PLoS Med..

[26] Kucharski, A. J. et al. On behalf of the CMMID COVID-19 working group, Effectiveness of isolation, testing, contact tracing, and physical distancing on reducing transmission of SARS-CoV-2 in different settings: a mathematical modelling study, *Lancet Infect. Dis.*10.1016/S1473-3099(20)30457-6 (2020). 10.1016/S1473-3099(20)30457-6 PMC7511527 32559451

[27] Bi Q (2020). Epidemiology and transmission of COVID-19 in 391 cases and 1286 of their close contacts in Shenzhen, China: a retrospective cohort study. Lancet Infect. Dis..

[28] Klepac, P. et al. Contacts in context: large-scale setting-specific social mixing matrices from the BBC Pandemic project. 10.1101/2020.02.16.20023754 (2020).

