# Supplementary material for: A population-based controlled experiment assessing the epidemiological impact of digital contact tracing
Source: Nat Commun. 2021 Jan 26;12:587. doi: 10.1038/s41467-020-20817-6 (PMC7838392; doi:10.1038/s41467-020-20817-6)
Supplement: Supplementary file 1 — Supplementary Information [file 41467_2020_20817_MOESM1_ESM.pdf]

Supplementary Information for A POPULATION-BASED CONTROLLED EXPERIMENT ASSESSING THE EPIDEMIOLOGICAL IMPACT OF DIGITAL CONTACT TRACING

Suppementary Note 1: RadarCovidPilot group

In alphabetical order, the RadarCovidPilot (RCP) group is formed by the following people:

Carlos Hugo Acosta[a], José Ignacio Aguillo[g], José Juan Alemán[a], Eva Elisa Alvarez-León[a], Alex Arenas[b], Carme Artigas[c], Borja Barroso[a], Manuela Battaglini[d], Fátima Bezares[a], Eleazar Jesús Borrego[a], Isidro Manuel Brito[a], Francisco Javier Darias [a], Marta Díaz[a], Laura Flores[c], Leticia Gómez[g], Jaime González[g], Santiago Graña[c], Miguel A. Hernán[e], Pablo A. Hernández[a], María Ángeles Jerez[a], Octavio Jiménez[a], José Fernández Lacasa[c], Lucas Lacasa[f,k], Raquel López[g], Paloma LLaneza[h], Maria Cristina Martín[g], Gloria Martinez[c], María Jesús Millán[a], Domingo Nuñez[a], Oriana Ramirez-Rubio[j], Pablo Rodríguez[l], Adriana Romaní[j], Javier Sánchez-Monedero[i], Berta Suárez-Rodriguez[j], Jesús Torres[c], Lucía Velasco[c], Borja Ventura[c].

[a]Dirección General de Salud Pública, Servicio Canario de la Salud, Gobierno de Canarias (Spain)
[b]Departament d'Enginyeria Informàtica i Matemàtiques, Universitat Rovira i Virgili, 43007 Tarragona (Spain)
[c]Secretaría de Estado de Digitalización e Inteligencia Artificial, Ministerio de Asuntos Económicos y Transformación Digital, Madrid (Spain)
[d]Transparent Internet, Tårup Bygade 30, DK-5370 Mesinge (Denmark)
[e]Department of Epidemiology, Harvard TH Chan School of Public Health, Boston, MA (USA)
[f]School of Mathematical Sciences, Queen Mary University of London, London E14NS (UK)
[g]User Experience, INDRA (Spain)
[h]Razona LegalTech, Madrid (Spain)
[i]School of Journalism, Media and Culture, Cardiff University, Cardiff CF101FS (UK)
[j]Centro de Coordinación de Alertas y Emergencias Sanitarias. Dirección General de Salud Pública, Calidad e Innovación. Ministerio de Sanidad (Spain).
[k]Instituto de Física Interdisciplinar y Sistemas Complejos IFISC (CSIC-UIB), Palma de Mallorca (Spain).
[l]Association for Computing Machinery (ACM).

Suppementary Note 2: Additional details on the app

Onboarding and presentation

The app onboarding is depicted in Supplementary Fig.1. This onboarding only shows up once (the first time the user opens the app)[1].

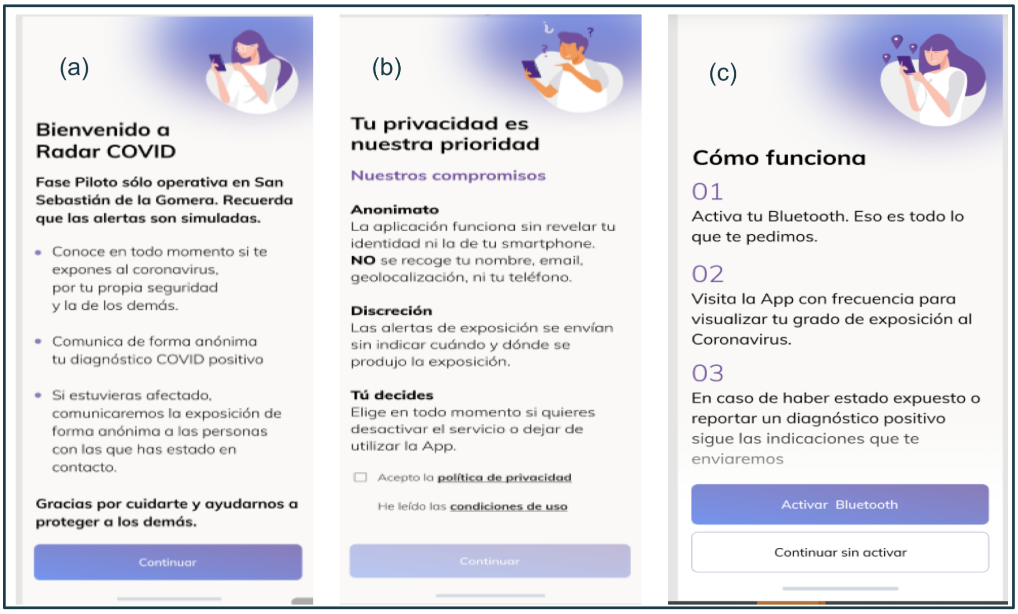

Supplementary Figure 1: **Onboarding.** Onboarding of the app, with three successive panels with succinct information.

After onboarding, the app only shows one panel on the status of the phone, with three possible states: low-risk state (no alarm has been triggered), high-risk state (alarm of a close-contact with a confirmed PCR+ individual has been notified), and confirmed PCR+ state (when the user has introduced the alphanumeric code given by primary healthcare to notify his matches they have been exposed). An illustration of the three states of this panel is depicted in Supplementary Fig.2.

Bluetooth parameters

In order to weigh in the exposure risk estimation both the exposure time and the distance, we apply a method where the exposure time is weighted by a factor that takes into account the Bluetooth attenuation range (as a proxy for distance, where the larger the attenuation, the farther away the two smartphones are to each other).

Initially we set a conservative bluetooth attenuation range $[53 - 60]$, meaning that any signal between two phones whose attenuation is below 53dB is giving a 100% weight, i.e. an exposure time of 15 minutes is traduced into an effective exposure time of 15 minutes. For signals whose attenuation range was somewhere between 53dB and 60dB, the effective exposure time was reduced by 50%, meaning that a true exposure time of 15 minutes at a distance which relates to an attenuation range of, say, 58dB, was traduced into an effective exposure time of 7.5min. Over 60dB, we assumed that the contact did not happen. Finally, a match happened when the

[1]Note that for the nationwide deployment, besides Spanish the app is available in English and several other co-official languages such as Catalan, Euskera, Galician

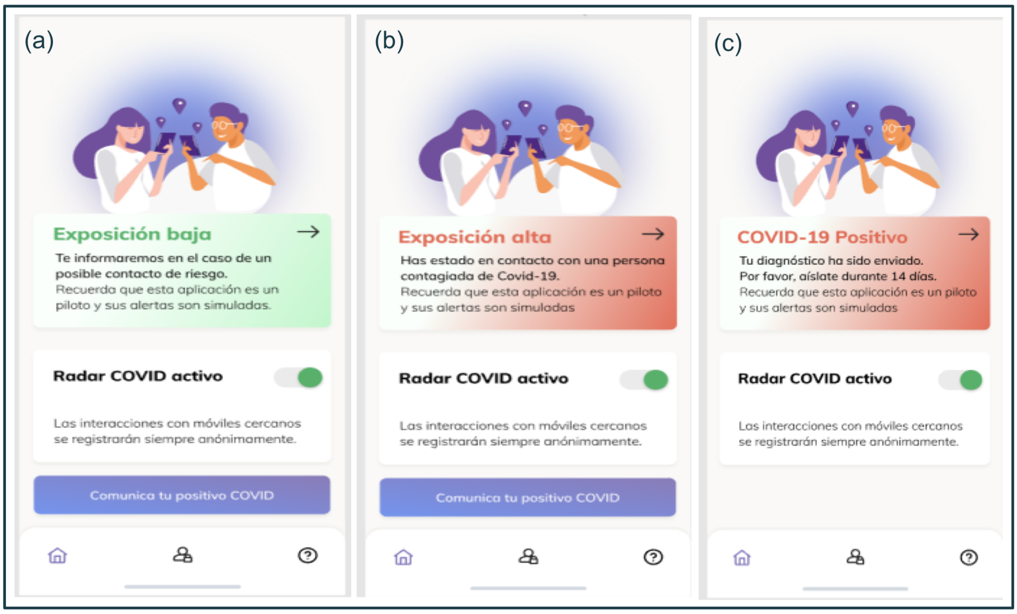

Supplementary Figure 2: **Dashboard.** The app single dashboard panel, showing the three possible states: low-risk (no alarm has been triggered), high-risk state (alarm of a close-contact with a confirmed PCR+ individual has been notified), and confirmed PCR+ state (when the user has introduced the alphanumeric code given by primary healthcare to notify his matches they have been exposed).

effective exposure time was over 15 min.

A key re-calibration was conducted in the 15th July, where less restrictive attenuation ranges were considered. As a matter of fact, experiments in lab conditions [1] suggested that the optimal attenuation range for a correct detection was not $[53-60]$, but $[63-74]$. Accordingly we proceeded to make an update of the weights. Due to a variety of reasons with DP3T protocol we were not able to tightly update the lower bound, so finally the 15th July recalibration the 50% weight was added to the range $[55-74]$:

- Below 55dB attenuation, the exposure time is multiplied by a factor 1,
- Between 55dB and 74dB the exposure time is multiplied by a factor 0.5,
- For any attenuation over 74dB the exposure time is multiplied by a factor 0, i.e. this contact is not recorded,
- A match is recorded if the resulting effective exposure time is over 15 min.

Note on cybersecurity

The app has undergone several security and failure detection checks by the National Cryptologic Centre (CCN).

Suppementary Note 3: Recruitment and communication campaign

The communication campaign was designed covering four different areas (see Supplementary Fig.3):

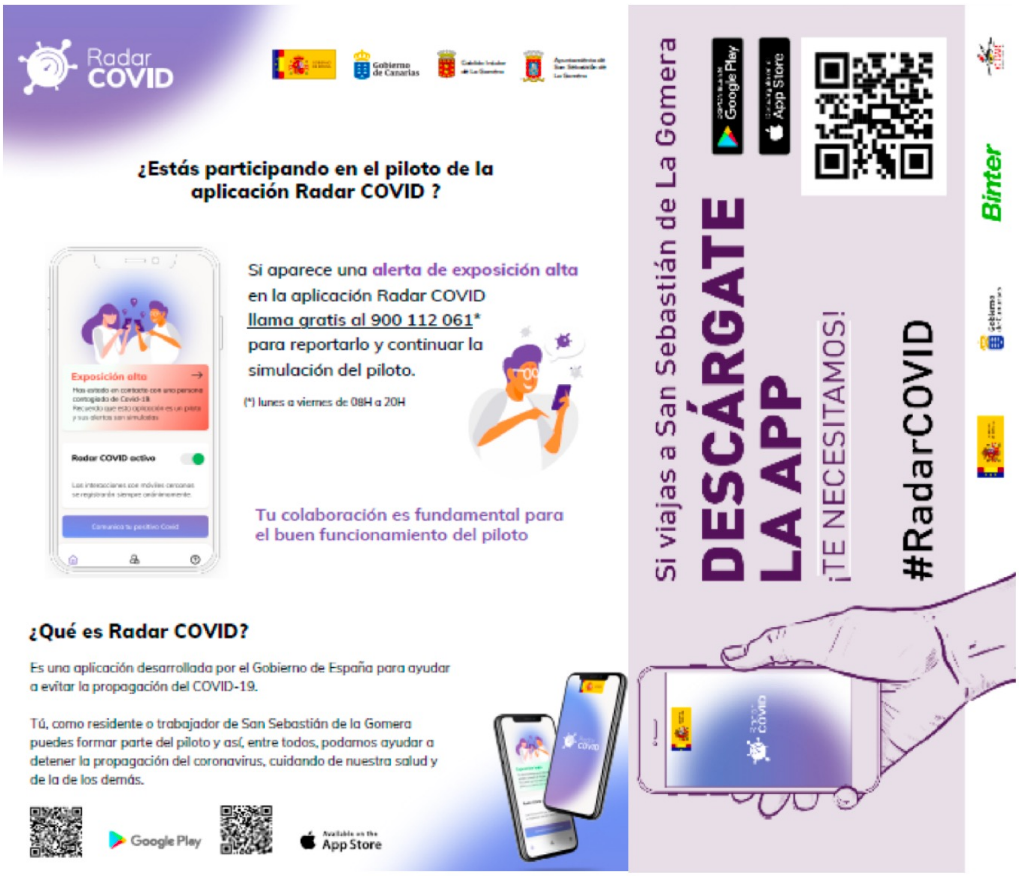

Supplementary Figure 3: **Marketing materials.** Examples of Marketing materials deployed in the recruitment campaign, targeting both citizens of San Sebastian de la Gomera as well as commuters using the Navieras (boats from Tenerife) and the regional airline Binter.

- Direct contact with civil servant cohort: Direct email and telephone recruitment was deployed with a range of civil servants from La Gomera, both from the town hall and the local healthcare system. A total of 758 people were contacted.

- Offline promotion and promoters: 12 marketing and recruitment officers were hired to conduct recruitment sessions where the agents could inform and help citizens with the process of downloading the app in their smartphones, as well as distribute marketing material. These promoters also collected feedback from citizens. They were actively engaging with citizens in their local stands, available between 30th June and 10th July, from monday to friday 10AM-1PM and 5PM-7.30PM in four different locations: (i) Plaza de Las Américas, (ii) Plaza de La Asunción, (iii) Mercado Municipal, and (iv) Centro de Salud de San Sebastián de La Gomera.

- ads on public transport: including regional airline Binter and Naviera boat Fred Olsen. An additional 4 promotion agents advertised the app in the Naviera that connects La Gomera with Tenerife between 11 and 15th July (i.e. before the Navieras simulated outbreak).

- an online campaign was also launched in social media and the app was available to download from the web of the Canary Islands government.

Suppementary Note 4: Data Collection

Data for this controlled experiment was collected through a variety of means, namely:

- **From promoters:** Daily records of participants recruited by promoters, who also collected data on DOB and genre (this information also allowed us to tactically decide when to move promoter's locations). Records of codes initially distributed to participants, including DOB and genre information of the users who voluntarily agreed to be primary case infections. Promoters also collected feedback on the app perception.

- **From call center:** A (free call) call center was deployed for the experiment, whose role was twofold: (i) serve as a contact point for participants to clarify any doubt they could have, and (ii) serve as the primary healthcare call center to which the users that were alerted by the app they were in close contact to a PCR+ individual had to make the follow-up call. This call center kept a daily record of calls and an additional questionnaire which includes (i) the date when the app notified the alert, (ii) if on that date the user recalls having a risk exposure (indicating awareness of exposure and whether the contact was with a stranger), (iii) the place where the user lives and (iv) DOB and genre.

- **From the app surveys:** the app offers the possibility of conducting a satisfaction survey where the user can provide feedback on various aspects.

- **From user tests:** in-depth interviews with 15 participants.

- **From the API and the Radar COVID cloud server**: (i) daily record of number of codes introduced in the app and (ii) number of alerts triggered (aggregated and anonymised, there is no possibility of tracing back an alert to a given simulated infection code), (iii) daily number of active apps.

From the app cloud server

Date	10/07	11/07	12/07	13/07	14/07	15/07	16/07	17/07	18/07	19/07	20/07	21/07	22/07
Outbreak size (number of codes that should be introduced in the app)	150			90		49		60					
Pre-symptomatic phase (days)	4			7		4		11					
Cumulated number of codes introduced in the app	104	108	111	154	159	185	193	233	235	236	250	251	251
Cumulated number of alerts triggered by the app	20	20	20	180	240	270	346	542	655	678	763	807	821
Cumulated calls to call center	2	2	2	19	25	28	37	51	51	51	74	80	81

Table 1: Summary of raw data, reporting some design specifications (number of days the voluntary participants remain in the pre-symptomatic phase before introducing the code, and designed size of the outbreak), and some data collected (cumulated daily number of codes introduced in the app, alerts notified by the app, and calls to call center. Date of different epidemic outbreaks is highlighted in gray.

A summary of quantitative data extracted both from the Radar COVID cloud server and call center statistics is depicted in table 1.

Online surveys

On relation to the online surveys, these are voluntary surveys available on the app, where the user was also allowed to only answer to a handful of questions. A preliminary question allows us to distinguish those participants that indeed live or work in San Sebastian de La Gomera, the municipality where the controlled experiment was conducted, from those that did not (either tourists, or people that downloaded the app from outside the municipality). The subsequent questionnaire of 17 questions covers aspects on app usability and app efficiency. The list of questions is the following:

- **app usability**: in a scale 0-10, where 0 is very bad and 10 is excellent, how would you mark:

  1. how easy was to install and use the app,
  2. how easy is to follow the instructions depicted in the app,
  3. the visual design of the app,
  4. the feeling of privacy and anonymity,
  5. the overall functioning of the app,
  6. the overall experience while using the app.
  7. Would you recommend a family member or a friend to use the app?

- **app efficiency** (where we can distinguish three roles: infected individual, user notified by the app as having been exposed to a high-risk contact with a PCR+ individual, rest of users). For the first role:

  8. Did you participate in the controlled experiment as an individual with a PCR+ role? [The user clicks YES]
  9. How easy was to introduce the code [0-10 scale]?
  10. How do you assess the information you received when you introduced the code [0-10 scale]?
  11. If you were given a chance, which form of notification would you choose [app alert / in person (medical doctor) / both options]?

  Now, for the second role:

  12. Did you receive an alert on the app? [The user clicks YES]
  13. How clear was the information received [0-10 scale]?
  14. If you were given a chance, which form of notification would you choose [app alert / in person (medical doctor) / both options]?
  15. Do you think the high-risk contact was with an acquaintance or with a stranger?

  For all users:

  16. Do you think this app can help to prevent disease spreading? [Y/N]
  17. Did you have any problem with the app that made its use difficult? [If responded Y to the last question] Briefly describe the issue [max 100 characters]

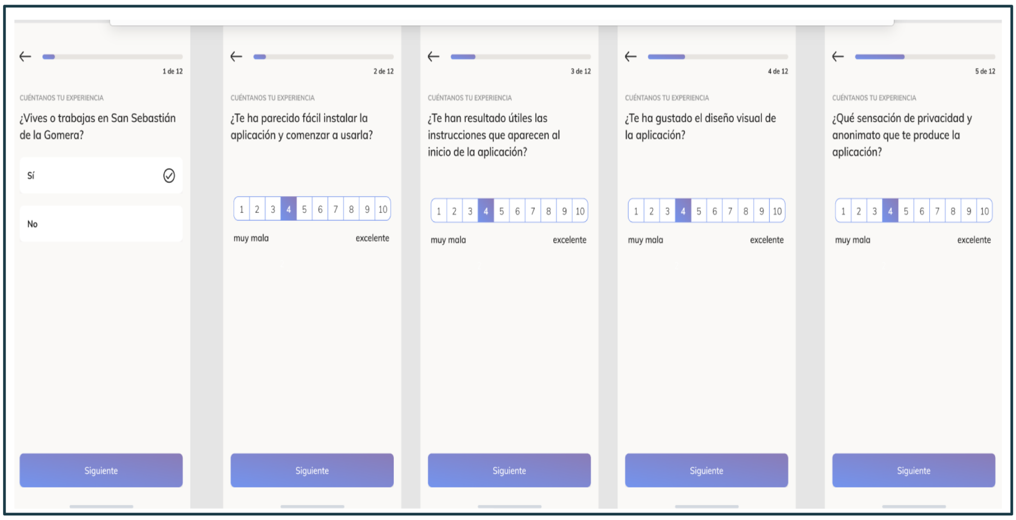

Supplementary Figure 4: Example of questions in the app survey.

An example of how the app survey looks like is depicted in Supplementary Fig.4.
We collected a total of 735 app surveys. Out of these, only 12% declared to be living or working in SS de la Gomera, the other 88% could include tourists and people that downloaded the app outside SS de la Gomera. We call this cohort "other". A total of 64 surveys were from users with PCR+ role (39 from SS de la Gomera, 25 other), 51 with a close-contact role (38 from SS de la Gomera, 13 other). Information retrieved from participants outside La Gomera were useful for feedback on usability, however it is difficult to interpret answers to Qs 9-15 as these people could either be people from other parts of Spain who therefore did not participate in the controlled experiment, or otherwise be tourists and daily commutters to SS de la Gomera who indeed participated in the experiment but just didn't live and work there. In any case, we have splitted results in two groups (from SS de la Gomera, or other). Questionnaire results are summarised in table 2, showing that usability is overall good. Interestingly, in the app surveys up to 29% of the close-contacts (question 15) of a PCR+ were with strangers for those participants that declared they were living or working in SS de la Gomera, and this percentage increased up to 39% for those that declared they didn't live or work there, suggesting that app detection of hidden transmission chains is significant.

in-depth interviews

We performed in-depth (45 min) interviews with 15 individuals: 11 of them participated in the controlled experiment covering the four age ranges considered (2 in the 18-30 yo, 4 in the 31-40 yo, 4 in the 41-60 yo, 1 in the >60 yo range), and 4 additional people which did not participate were also interviewed (0/1/2/1). Most of the discussion versed on usability and data was either qualitative or similar from previous data extracted from online surveys, so we don't report here the specifics. There are however a subset of questions that brings additional information, which we report here. As a caveat, note that the sample size of in-depth interviews is too small (15 interviews) for the result to be representative.

1. To the question "I will keep on using the app once it is launched officially", all 15 participants responded affirmatively.

2. To the statement "Most of the population will download the app", in a 1-10 scale where 1 stands for 'I disagree' and 10 stands for 'I agree', the average mark was 6.9/10. Invoking a wisdom of the crowds argument, this would suggest a prospective adoption of about 69%.

Question	Result
1	8.1/10 in SS de la Gomera, 8.8/10 other
2	7.9/10 in SS de la Gomera, 8.3/10 other
3	8.2/10 in SS de la Gomera, 8.3/10 other
4	7.8/10 in SS de la Gomera, 8/10 other
5	6.9/10 in SS de la Gomera, 7.6/10 other
6	7.3/10 in SS de la Gomera, 7.6/10 other
7	7.8/10 in SS de la Gomera, 8.5/10 other
9	7.3/10 in SS de la Gomera, 7.6/10 other
10	7.6/10 in SS de la Gomera, 7.5/10 other
11	app
13	6.5/10 in SS de la Gomera, 7/10 other
14	app
15	29% stranger in SS Gomera, 39% stranger other
16	82% YES in SS Gomera, 82% YES other
17	38% YES in SS Gomera (mainly related to app updates), 17% YES other

Table 2: Summary of results of the app surveys. We have uncoupled results from those that declared they were living and working in SS de la Gomera from those in 'other'.

3. To the statement "Those close-contacts notified by the app will comply and make a follow-up call to the suggested call-center", the average mark was 8.3/10, i.e. a substantial agreement. This is in stark contrast with the actual number of follow-up calls in the experiment, that was around 10%. We speculate that, perhaps, the difference stems in the fact that close-contacts know that infections are simulated, so once they are alerted by the app they have been exposed, they might not follow-up as they don't have the incentive of e.g. receiving a PCR test. However, as noted above, the sample size of the in-depth interview is too small to draw conclusions.

4. To the statement "those individuals who have been given a positive PCR will introduce the code in the app", the mark was 8.4/10.

5. When asked about the probability of close-contacts to be with strangers, in a 1-10 scale where 1 stands for 'stranger' and 10 stands for 'acquaintance', the mark was 6.1/10, suggesting that the estimate of stranger close contacts is about 40%, on good agreement to the app survey results performed on tourists and commuters.

A note on privacy

Since infections are simulated, all app alerts triggered after participants introduced the codes are in that sense also simulated –in a nutshell, no real epidemic outbreak took place, we explored how a real epidemic outbreak would have propagated and how many close-contacts would have been detected. In order to assess the efficiency of the app, in this experiment we also were able to measure an aggregated and anonymous metric quantifying the total daily number of those alerts. Note that such metric is made available during the controlled experiment for the purpose of validating the efficiency of the app, and collected for research purposes as part of the privacy policy. All communications with the participants of the experiment (through promoters, app surveys, in-depth interviews, etc) followed the adequate privacy policy.

Suppementary Note 5: Estimating adoption

During the time over which the experiment was conducted, over 11 thousand direct downloads from the Apple and Google servers took place. In absolute terms, that would be above 100% adoption! (San Sebastian de la Gomera hovers around a population of 10,000) Now, while during the experiment the app was designed to work only in San Sebastian de La Gomera, unfortunately it was also available for download nationally, and since the app is privacy-friendly we don't have any geolocalisation data. While there was no incentive or advert to download this app outside the controlled experiment, we cannot rule out that a percentage of those that downloaded the app from the Apple and Google stores did so from outside San Sebastian de la Gomera, e.g. curious individuals which simply wanted to see what the app looked like. Accordingly, a different way of estimating adoption had to be sought. In what follows we provide estimates based on a different number of assumptions.

Our first estimate (probably overconservative) is based on counting only those downloads that we can absolutely guarantee were performed by participants which were in San Sebastian de la Gomera. A total of 924 downloads were performed by street promoters during promotional sessions, i.e. people who interacted with street promotors during recruitment sessions in different geographical locations of San Sebastian de la Gomera. 55% are women, and the age distribution of these 924 participants is plotted in Supplementary Fig.5.

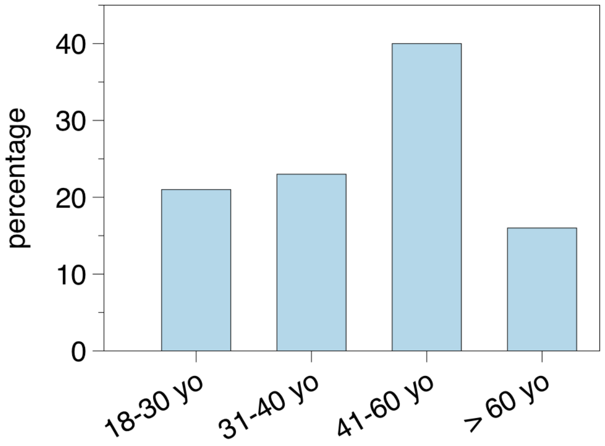

Supplementary Figure 5: **Age distribution.** Age distribution across the cohort of 924 participants recruited by the street promoters.

On addition to these, a total of 241 were downloads from a local weblink from the Canarian government, for a total of 1165 downloads, i.e. an initial 11% adoption.

On addition to these, our second estimate adds a total of 758 civil servants from La Gomera, who were directly requested by the government to downloaded the app (however there was no doublecheck to certify they did comply). These civil servants were not only instructed to download the app, but were also explicitly asked to recruit family members and friends, and for that sake they were given member-get-member invitations (6 per individual). We conservatively assumed that each civil servant on average recruited 3 other people. What would be the likelihood that both the civil servants and their recruitees actually downloaded and activated the app? Probably such likelihood is larger for the civil servants and slightly smaller for their recruitees, but otherwise is unknown. As a fair approximation, we propose to use the

actual compliance we have observed in this experiment: 64% (see below). That means that an additional $(758 + 758 \cdot 3) \cdot 0.64 = 1940$ downloads, i.e. a total of $1940 + 1165 = 3105$ downloads, or a 31% adoption.

So far we have not included any download incurred from spontaneous adoption, i.e. downloads from the Apple and Google servers from people that saw some kind of marketing information and did not require the help of any promoter, word of mouth from those recruited from street promoters, etc. We don't know that percentage, but we know that the Apple and Google server had over 61k downloads during the weeks of the experiment! Let's again be conservative, and let us assume that spontaneous adoption constituted a tiny 2% of the population. That would add about 200 downloads, for a total of 3305, or a 33% adoption. This is the estimation we use in the main part of the manuscript. We consider it to be a conservative estimate, and larger percentages of spontaneous adoption would yield substantially larger adoption percentages. For instance, from the in-depth interviews (small sample size) we find an estimate of 69%.

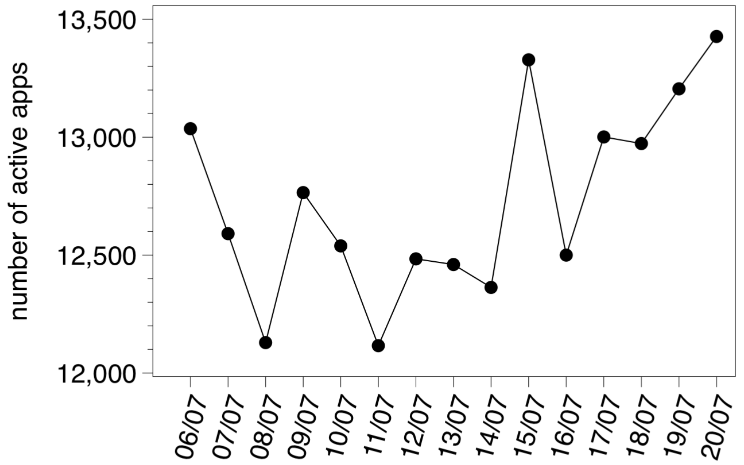

Supplementary Figure 6: **Active apps.** Number of active apps over the timeline of the controlled experiment, suggesting a high adherence.

As we have already argued, it is not possible to accurately estimate how many downloads out of the 61k were performed by participants in the experiment. Since the app and the experiment was heavily advertised in La Gomera (and seldom outside Canary Islands), probably the vast majority of the participants that spontaneously wanted to download the app went directly the Apple Store/Google Play, whereas curious Spanish citizens from other places of Spain might have needed to investigate on Google –to begin with, the name of the app–. Anecdotally, using Google Trends we found that during the weeks of the experiment, while the term "app covid" was indeed queried in Google across several Spanish autonomous communities, the Canary Islands ranked very high in the frequency of the query –although the ranking was somewhat unstable with respect to changes in the dates, what suggests that overall queries are low–. This is an indirect evidence of additional spontaneous adoption (e.g. people that wanted to download the app but didn't know the name of the app and thus Googled it beforehand). Note that in Google query we use "app covid" instead of "radar covid", since we argue those citizens that already know the name of the app don't need to query it on Google, they can directly go to the Applestore or Google play.

Let us now comment further on the relation between adoption, recruitment and communication campaigns.
First, we can conclude that the communication and recruitment campaign was not only successful,

but probably instrumental for attaining the adoption we reached. This means that any successful national deployment of the app might need equivalent communication campaign to be deployed to scale.
Second, we should distinguish the overall adoption (the total number of people that downloaded the app) from adoption density, which is a local measure which can vary from region to region. While in our experiment both measures are essentially related because San Sebastian de la Gomera is a small city, in a national deployment one could have different scenarios. For instance, we could envisage a scenario with a reasonable overall adoption but small, even subthreshold, adoption density (e.g. if the adoption is uniform across the nation, locally the density could be subthreshold such that the detection rate would drop). We could also have a different scenario where the overall adoption is exactly the same as in scenario 1, but with heterogeneous adoption density (i.e. regions with high adoption density, and regions with low adoption density). If we compare these two scenarios, from the epidemiological side, the second is probably to be preferred. This difference between overall adoption vs adoption density speaks to the fact that communication campaigns should be deployed at a national scale (aiming to boost overall adoption) and at a regional one (more targeted recruitment campaigns, designed to boost local adoption densities).

Suppementary Note 6: Estimating adherence

To estimate adherence, we look at the number of apps which remain active for the whole duration of the controlled experiment, i.e. with the capacity of receiving alert notifications. This number is fairly constant, fluctuating in the range 12k-13.5k, see Supplementary Fig.6. We conclude that adherence was high, at least during the whole experiment. We don't have additional data that could indicate whether adherence is maintained in the longer term.
Some additional, indirect evidence on adherence can be extracted from the results of both app surveys and in-depth interviews. From 735 app surveys, 82% concluded that the app was a useful tool, and the question "I will recommend friends and family members to download and use Radar COVID" was given 7.8/10 marks. From the list of in-depth interviews (15 surveys) the question "I will keep on using the app when it is officially launched" reaches full marks (although note there is possibly a strong selection bias here, as those people willing to make an in-depth interview are people which are engaged with the app to begin with).

Suppementary Note 7: Estimating compliance

Compliance is simpler to estimate, and it is based on two factors: the percentage of codes of primary infections which were actually introduced, and the percentage of codes of secondary infections which were introduced. A total of 349 codes where initially distributed (primary infections), and out of these 213 codes were logged in, i.e. a 61% primary infection compliance. Additionally, a total of 43 codes were issued on relation to secondary infections, and 38 of these were introduced, i.e. a 88% secondary infection compliance. In order to avoid needing to interpret which of the two compliances better represent total compliance, we simply aggregated the results and computed the total number of codes which were introduced over the total number of codes which were issued, i.e.

$$\frac{213+38}{349+43} = 0.64,$$

i.e. 64% compliance.

Suppementary Note 8: Estimating Turnaround time

Out of the 349 initial codes (corresponding to the different outbreaks), 300 were assigned to the downtown outbreaks (10th, 13th and 17th July) and those were distributed on the 6th of July to the voluntary participants, along with specific instructions on the day where these people should introduce the code in the app to notify their contacts. This allowed to build up different pre-symptomatic windows (4 days in the case of the first outbreak, 7 days in the case of the second, and 11 days in the case of the last outbreak). The 49 remaning codes were distributed on the 11th of July and corresponded to the outbreak of the Navieras (15th July), thus allowing for a 4 day pre-symptomatic phase.
Some primary infected individuals never introduced the code (about 136 out of 349). Of those that complied, 193 introduced it in the correct date. Now, when a code is introduced in between two epidemic subsequent outbreaks, we cannot discern whether that code is a code belonging to the preceding epidemic outbreak that was introduced late, or a code belonging to the subsequent epidemic wave which is introduced early. Accordingly, to estimate the % of those primary cases which are compliant with the app that introduce the notification *on time*, we only consider data from the first epidemic outbreak (10th July), where the aforementioned source of ambiguity is reduced. In this outbreak a total of 150 codes should be introduced (see table 1). Assuming a homogeneous 64% compliance rate, we thus expect that 96 codes should be introduced. A total of 84 codes where indeed introduced precisely on the 10th of July (86%), but an additional 20 codes (20.5%) were introduced a few days beforehand. These extra 20.5% could be related to users that disregarded the date and just introduced the code straight away at the beginning of the experiment. We then assume that the first 20 codes where early codes from all three epidemic outbreaks (10th, 13th, 17th July), and for simplicity we assume a uniform sampling meaning that out of these 20 codes those related to the first epidemic outbreak are the $[150/300] \cdot 100 = 50\%$, i.e. about 10 codes. Accordingly, in the first epidemic about $84+10 = 94$ codes were introduced on time, out of an estimated total of 96 codes, i.e. about 98%.

Outbreaks	10th July	13th July	Naviera 15th July	17th July
Turnaround time from code notification of primary case to call center follow-up call of secondary-case (days)	2.96	1.35	1.92	3.18

Table 3: Average turnaround time from code notification to follow-up call for the four epidemic outbreaks simulated in the experiment.

Additionally, the second factor to be taken into account is the time taken between a primary case informs the app (i.e. introduces the code), and a close-contact who is alerted by the app makes a follow-up call to the primary healthcare call center. Note that we cannot trace back any given alert to its origin primary case, but still we can make the assumption that all outbreaks are independent and there is no overlap, in such a way that we can obtain average estimates of the time between code notification and call to call centers. In table 3 we provide such average estimates, for each epidemic outbreak. Averaging over outbreaks, the average turnaround time across all outbreaks is 2.35 days.

Incidentally, note that the specific implementation of the app specifies the time taken between a primary case introduces a code in the app and the app alerts her close-contacts. This is not necessarily instantaneous, for instance under the implementation used during the controlled

experiment, the app only sent alerts twice a day. Assuming that codes were introduced at a somewhat uniform rate, then a very crude estimate would suggest an average time of 6 hours (i.e. 0.25 days) between introducing the code and receiving the alerts. This delay of 0.25 days could easily be discounted from the average turnaround time (2.3 days) simply by changing the app implementation and allowing it to be updated and send alerts more often.

Suppementary Note 9: Estimating overall detection

To adequately measure the daily average rate of matches per primary infection, we adopt the following heuristic: there exists a lag between the user introducing a code and the alert notification (as the app only performs such notifications twice a day), so we assume that 50% of the daily alerts (matches) in a given day correspond to codes notified that day, and 50% correspond to codes notified the day before. The resulting smoothed average rate is reported in Supplementary Fig.7. Incidentally, note that while different epidemic outbreaks are in principle non-overlapping, late introductions of codes can yield effective overlapping. This is aggravated by the fact that different outbreaks impose different pre-symptomatic windows, in such a way that the specific daily average of matches per infection is not totally interpretable or comparable. This issue notwithstanding, when we plot such average over time we can see how the bluetooth re-calibration has a net and measurable effect on boosting detection.

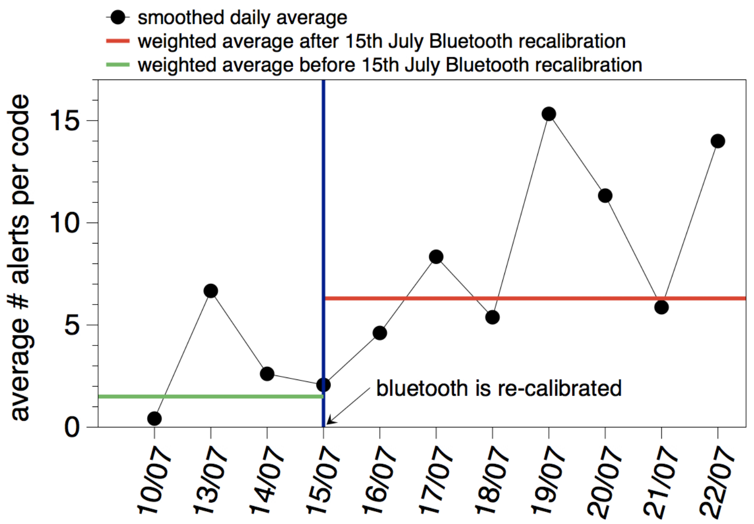

Supplementary Figure 7: **Alerts per code.** Estimated smooth daily average of digitally-traced contacts per primary infection. For comparison, we also show the weighted average of 6.3 contacts per primary infection. We can see how bluetooth re-calibration (15th July) boosted detection.

Of course, the total average is not just a point average of the daily rates, since two days can have substantially different raw numbers and this needs to be weighted in. We therefore define the *overall detection rate* as the accumulated number of alerts over the accumulated number of codes introduced in the app within a certain time window. Note that even if the phones of two individuals who are close-contacts might have different matches over the course of several days, our metric only counts such interactions as one notification (one alert).
Here we consider two time windows, related to the two different Bluetooth parametrisations that were considered in the experiment: before 15th July and after 15th July. Before 15th July, the resulting overall detection rate was about 1.5 matches per code (240 alerts and 159 codes introduced before 15th July). This number was probably an underestimation of the actual

close-contacts per primary infection, and indeed after 15th July recalibration, in the second time window we find an overall detection ratee of about 6.3 matches per code (581 alerts and 92 codes introduced after 15th July).

We can compare such overall detection rate of 6.3 close-contacts per primary infection with an equivalent manual detection rate, i.e. the average number of close-contacts which can be traced manually. The local health authorities of the Canary Islands (some of whom are co-authors of this paper) certify that, on average, over the course of the experiment the average number of manually traced contacts was about 3.5. On the other hand, we do not have *average* numbers for Spain, only *medians*. In July, the median of the manual detection rate was 3 in Spain. We should make some important comments on relation to these comparisons. First, note that reaching an overall (digital) detection rate of 6.3 close-contacts per primary case is an indirect evidence that the adoption density was significant. To reach similar levels if the app is deployed nationally, then one should reach similar adoption density. Second, note that the comparison with manual tracing was made when all Spain was at very low incidence rates, and manual tracers were not overwhelmed. In an epidemic outbreak however, in order for the manual detection rate to be maintained to 3-3.5, this requires a significant surge of manual tracers. Past experience suggests that flexibly hiring the sufficient number of manual tracers is not always possible. When there is a shortage of manual tracers, manual detection rate will drop significantly. Since DCT is, a priori, independent of manual tracers, we hypothesise that, with a good adoption density, the detection rate of the app could be even higher than twice-as-much as the manual detection rate during epidemic outbreaks.

Suppementary Note 10: Estimating hidden detection

We base the detection of contacts between strangers (leading to manually untraceable transmission chains) on two sources of data: (i) information reported to the call center by those users that followed-up an app alert with a call to the primary healthcare call center, and (ii) online surveys.
23% of those users that contacted the call center stated that the alert notification they received was probably related to a close-contact with a stranger, whereas this percentage increased to 29% and 39% in the case of online surveys (which is a different sample). These numbers should be treated with caution as they are solely based on questionnaires and surveys.

References

[1] Data obtained in the Dutch Fieldtest Vught – 8 june 2020. Private communication.
