# Supplementary material for: A population-based controlled experiment assessing the epidemiological impact of digital contact tracing
Source: Nat Commun. 2021 Jan 26;12:587. doi: 10.1038/s41467-020-20817-6 (PMC7838392; doi:10.1038/s41467-020-20817-6)
Supplement: Supplementary file 2 — Reporting Summary [file 41467_2020_20817_MOESM2_ESM.pdf]

nature research

Corresponding author(s): Lucas Lacasa

Last updated by author(s): Oct 30, 2020

Reporting Summary

Nature Research wishes to improve the reproducibility of the work that we publish. This form provides structure for consistency and transparency in reporting. For further information on Nature Research policies, see our Editorial Policies and the Editorial Policy Checklist.

Statistics

For all statistical analyses, confirm that the following items are present in the figure legend, table legend, main text, or Methods section.

n/a	Confirmed	
☐	☒	The exact sample size (*n*) for each experimental group/condition, given as a discrete number and unit of measurement
☐	☒	A statement on whether measurements were taken from distinct samples or whether the same sample was measured repeatedly
☒	☐	The statistical test(s) used AND whether they are one- or two-sided<br>*Only common tests should be described solely by name; describe more complex techniques in the Methods section.*
☒	☐	A description of all covariates tested
☒	☐	A description of any assumptions or corrections, such as tests of normality and adjustment for multiple comparisons
☒	☐	A full description of the statistical parameters including central tendency (e.g. means) or other basic estimates (e.g. regression coefficient) AND variation (e.g. standard deviation) or associated estimates of uncertainty (e.g. confidence intervals)
☒	☐	For null hypothesis testing, the test statistic (e.g. *F*, *t*, *r*) with confidence intervals, effect sizes, degrees of freedom and *P* value noted<br>*Give P values as exact values whenever suitable.*
☒	☐	For Bayesian analysis, information on the choice of priors and Markov chain Monte Carlo settings
☒	☐	For hierarchical and complex designs, identification of the appropriate level for tests and full reporting of outcomes
☒	☐	Estimates of effect sizes (e.g. Cohen's *d*, Pearson's *r*), indicating how they were calculated

Our web collection on statistics for biologists contains articles on many of the points above.

Software and code

Policy information about availability of computer code

Data collection	Data in the controlled experiment was collected using the app Radar Covid, see code https://github.com/radarcovid
Data analysis	N/A

For manuscripts utilizing custom algorithms or software that are central to the research but not yet described in published literature, software must be made available to editors and reviewers. We strongly encourage code deposition in a community repository (e.g. GitHub). See the Nature Research guidelines for submitting code & software for further information.

Data

Policy information about availability of data

All manuscripts must include a data availability statement. This statement should provide the following information, where applicable:

- Accession codes, unique identifiers, or web links for publicly available datasets
- A list of figures that have associated raw data
- A description of any restrictions on data availability

All data analysed during this study are included in this published article (and its supplementary information files).

Field-specific reporting

Behavioural & social sciences study design

All studies must disclose on these points even when the disclosure is negative.

Study description	Mixed-methods case study (controlled experiment) where a sample of the population of San Sebastian de la Gomera (population 10,000), after a communication campaign, downloaded the digital contact tracing app RadarCovid, and used it in a controlled environment. Fictitious outbreaks were simulated and the study analysed how the participants that were given codes to simulate index cases notified that in the app, how that triggered secondary-case notifications, and how the participants that received the notifications contacted the call center to follow up.<br>app-based data was completed with questionnaires
Research sample	Roughly 3,000 citizens of San Sebastian de la Gomera (Spain) downloaded the app and engaged with it. The exact number cannot be known as app downloads were voluntary. The age demography of a sample of 924 participants (the ones recruited by street promoters) is displayed in Supplementary Figure 5: 18-30yo (21%), 31-40 yo(22%), 41-60 yo (40%), >60 yo (17%)
Sampling strategy	Participants were recruited via different pathways: street promoters, direct recruitment of public workers, etc. A detailed estimation of the sample size is thoroughly provided in the Supplementary Information file (section 5).
Data collection	pen & paper, app, telephone questionnaires, app questionnaires were used to collect data. The whole data collection process is described in the Supplementary Information pdf.
Timing	the experiment took place over the month of July 2020 (4 weeks). No gap. Full details are available in the SI pdf.
Data exclusions	no data were excluded.
Non-participation	out of the total pool of about 10,000 citizens, we were able to recruit about 3,000. Participation was voluntary.
Randomization	participants were not allocated to experimental groups.

Reporting for specific materials, systems and methods

We require information from authors about some types of materials, experimental systems and methods used in many studies. Here, indicate whether each material, system or method listed is relevant to your study. If you are not sure if a list item applies to your research, read the appropriate section before selecting a response.

Materials & experimental systems

n/a	Involved in the study	
☒	☐	Antibodies
☒	☐	Eukaryotic cell lines
☒	☐	Palaeontology and archaeology
☒	☐	Animals and other organisms
☐	☒	Human research participants
☒	☐	Clinical data
☒	☐	Dual use research of concern

Methods

n/a	Involved in the study	
☒	☐	ChIP-seq
☒	☐	Flow cytometry
☒	☐	MRI-based neuroimaging

Human research participants

Policy information about studies involving human research participants

Population characteristics	see above.
Recruitment	Participants were recruited via different pathways: street promoters, direct recruitment of public workers, etc. A detailed estimation of the sample size is thoroughly provided in the Supplementary Information file (section 5). Since participation in the study was voluntary, The recruited participants might be more prone to engage with the app than a general citizen
Ethics oversight	Public Health General Directorate, Ministry of Health, Government of Spain

Note that full information on the approval of the study protocol must also be provided in the manuscript.
